# Oral manifestations in patients with coronavirus disease 2019 (COVID-19) identified using text mining: an observational study

**DOI:** 10.1038/s41598-023-44784-2

**Published:** 2023-10-18

**Authors:** Sandra Guauque-Olarte, Laura Cifuentes-C, Cristian Fong

**Affiliations:** 1 Faculty of Dentistry, Universidad Cooperativa de Colombia, Envigado, Colombia; 2 Faculty of Dentistry, Universidad Cooperativa de Colombia, Pasto, Colombia; 3 Faculty of Medicine, Universidad Cooperativa de Colombia, Santa Marta, Colombia

**Keywords:** Data mining, Dentistry

## Abstract

Text mining enables search, extraction, categorisation and information visualisation. This study aimed to identify oral manifestations in patients with COVID-19 using text mining to facilitate extracting relevant clinical information from a large set of publications. A list of publications from the open-access COVID-19 Open Research Dataset was downloaded using keywords related to oral health and dentistry. A total of 694,366 documents were retrieved. Filtering the articles using text mining yielded 1,554 oral health/dentistry papers. The list of articles was classified into five topics after applying a Latent Dirichlet Allocation (LDA) model. This classification was compared to the author's classification which yielded 17 categories. After a full-text review of articles in the category “Oral manifestations in patients with COVID-19”, eight papers were selected to extract data. The most frequent oral manifestations were xerostomia (n = 405, 17.8%) and mouth pain or swelling (n = 289, 12.7%). These oral manifestations in patients with COVID-19 must be considered with other symptoms to diminish the risk of dentist-patient infection.

## Introduction

The Coronavirus Disease 2019 (COVID-19) is caused by the SARS-CoV-2 virus that can infect the upper and lower respiratory tract^1^. The oral cavity is one of the entrances of SARS-CoV-2 into the body^2–4^. The infection of this structure can initiate the development of various oral manifestations such as xerostomia and dysphagia^2–4^. The disease can remain asymptomatic or cause severe pathology^5^ (pneumonia, dyspnea, organ dysfunction) and death^1^. As of 27 August 2023, more than 770 million COVID-19 cases have been confirmed, and over 6.9 million deaths have been reported (WHO, https://www.who.int/publications/m/item/weekly-epidemiological-update-on-covid-19---1-september-2023).

Between December 2019 and May 2023, a total of 362,313 publications related to COVID-19 were published (PubMed). To make COVID-19-related literature more accessible, biomedical literature databases such as the open-access COVID-19 Open Research Dataset (CORD-19)^6^ and the NIH COVID-19 Portfolio (https://icite.od.nih.gov/covid19/search/) were created. The Allen Institute for Artificial Intelligence created the CORD-19 in collaboration with the National Institutes of Health and the White House Office of Science and Technology Policy, among others. In the CORD-19, the PDF documents are converted into machine-readable JSON files that can be manipulated using programming languages. CORD-19 had 694,366 records by December 2021. The CORD-19 final release was on June 2, 2022.

Data mining enables extracting information from large amounts of text to be analysed using text mining methods. Text mining facilitates information extraction, categorisation, grouping, trend analysis and visualisation^7^. The goal is to focus information searches to remove noise^8^ and to identify hidden knowledge in the literature^9^. Zengul et al. used text mining to classify literature from the NIH COVID-19 Portfolio. They found several topics of COVID-19 research, such as patient care and outcomes, epidemiologic modelling, mental health and detection^10^. Tandan et al. used data mining to identify the common pattern of symptoms in patients with COVID-19^7^. Reddy et al*.* developed a biomedical platform to collect data on COVID-19 clinical risks. The use of this platform revealed the difficulty of extracting relevant clinical information through text mining and the need for feedback from experts in the field to obtain reliable results^9^.

Therefore, this study aimed to identify oral manifestations in patients with COVID-19 using text mining to facilitate extracting relevant clinical information from a large set of publications. Our study identified frequent COVID-19 oral manifestations that must be considered with other symptoms to diminish the risk of dentist-patient infection.

## Methods

This is an observational and descriptive study.

### Acquisition of data and text mining

A list of documents available in the CORD-19 database on January 31, 2022, was downloaded using the Kaggle notebook “Summary page COVID-19 risk factors” (https://www.kaggle.com/mlconsult/summary-page-covid-19-risk-factors). The notebook uses Pandas” built-in search technology. The notebook’s Python code was modified to find relevant articles by following this inclusion criteria: COVID-19 in abstract (‘COVID’, ‘-cov-2’, ‘cov2’, ‘ncov’, coronavirus), date of publication (January 1, 2020, and December 31, 2021), source of document (Elsevier, Medline, and PMC), and language (English, French, and Spanish). These were the exclusion criteria: preprints, reviews, communications, letters to editors, and documents lacking abstracts.

We search for keywords associated with oral health/dentistry. The search keywords were “buccal”, “dental”, “dentistry”, “dentist”, “odontology”, “oral” and “stomatognathic”. A search per keyword in the abstracts was performed independently and merged. Duplicate documents by title or abstract were removed. The notebook also scans the abstracts to print sentences (excerpts) containing the keywords to identify relevant articles rapidly. Finally, a text file containing “author”, “doi”, “title”, “abstract”, “excerpt”, “source”, “link”, and “publish time” was obtained. To curate the list of articles obtained, a text mining context analysis was performed to detect non-relevant (exclusion) keywords.

### Curation of the list of articles

To extract from the text file only articles related to COVID-19 and oral health/dentistry, we used the “Mining COVID-19 scientific paper” notebook with modifications, which is available on Kaggle (https://www.kaggle.com/mobassir/mining-covid-19-scientific-papers). The text file was uploaded to the notebook. Stop words (commonly used words, usually articles, that search engines are programmed to ignore) were removed from the abstract sentences. Stop words commonly found in scientific publications (such as “objective”, “methods” and “conclusions”) were also eliminated. Using the Gensim Python library, a 5-g (five-word combination) was created^11^^,^ and 494 “exclusion keywords” were identified. After visually scanning that the abstracts containing those “exclusion keywords” were not relevant, Python code was used to exclude them from the text file.

### Classification of articles

The next step of the text mining analysis was to use a Latent Dirichlet Allocation (LDA) model to the abstracts to classify the list of articles curated into topics. LDA is a probabilistic dimensionality reduction technique that can classify a document into two or more mutually exclusive classes or topics based on the frequency of the document's words. A distribution of words characterises each topic. The topic probabilities provide an explicit representation of a document^12^. The model assigned a dominant topic to each document and its percentage contribution,thus, a corpus of documents can be classified into topics depending on their subject.

### Author´s review of documents

**Figure 1:**
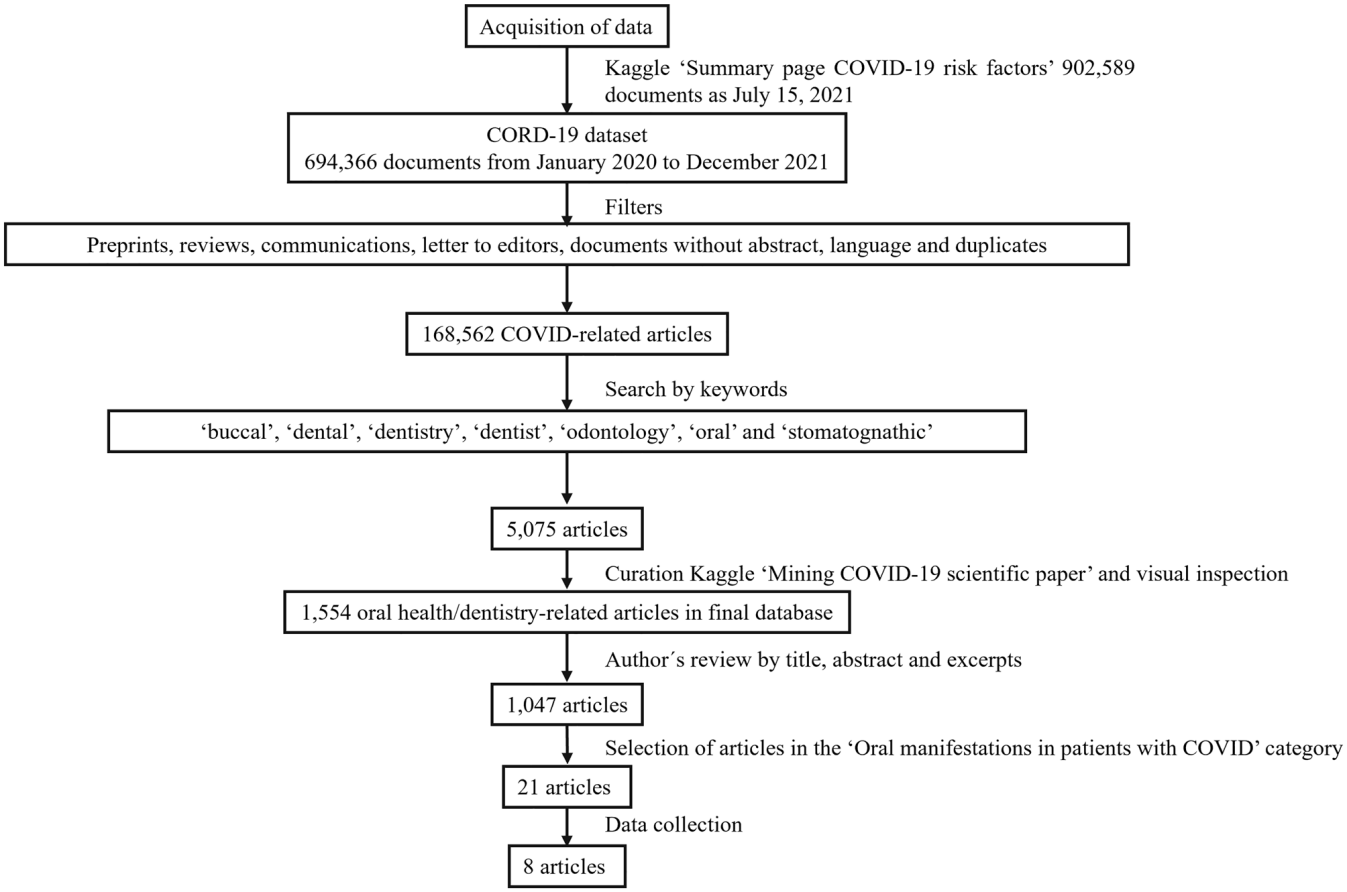
Workflow of the analysis. The documents available in the COVID-19 Open Research Dataset (CORD-19) were filtered using the “Summary page COVID-19 risk factors” and the “Mining COVID-19 scientific paper” notebooks available on Kaggle (https://www.kaggle.com/mlconsult/summary-page-covid-19-risk-factors and https://www.kaggle.com/mobassir/mining-covid-19-scientific-papers, respectively). The text mining classification of the documents was compared to the author's classification of the list based on title, abstract and excerpt. Finally, eight papers in the category “Oral manifestations in patients with COVID-19” were selected after full-text review to extract data.

To determine if the text mining process could classify the articles in the same categories that an expert would do, the authors reviewed each article title, abstract and excerpt in the curated text file to classify them. Finally, one category (“Oral manifestations in patients with COVID-19”) was selected for full-text review, and a database was created to summarise the results. The study workflow is summarised in Fig. 1.

### Ethics approval

Due to the nature of the study based on text mining, Ethical Approval was not required.

## Results

Between January 1, 2020, to December 31, 2021, the CORD-19 dataset contained 694,366 documents. Only 1,554 were oral health/dentistry related, even though the oral cavity is an entrance and reservoir of the SARS-CoV-2^13,14^ and the virus causes oral lesions^15–17^. The main subjects of the documents retrieved cover biosafety and changes in dental practice during the pandemic, the psychological implications for dentists and patients and the affectation of dental education during the COVID-19 pandemic.

Before downloading data and using Python commands, we removed 163,685 preprints, 361,713 duplicate documents based on title and 406 duplicates based on the abstract. Zero records had missing abstract. A keyword search was performed on the remaining 168,562 documents, yielding 5,075 articles that were downloaded: 42 for “buccal”, 1,294 for “dental”, 433 for “dentistry”, 797 for “dentist”, 17 for “odontology”, 2,489 for “oral” and 3 for “stomatognathic”. The remaining articles were written in English, French and Spanish and were included in further analysis. A file containing the 5,075 documents is available in Supplementary Table 1.

**Figure 2:**
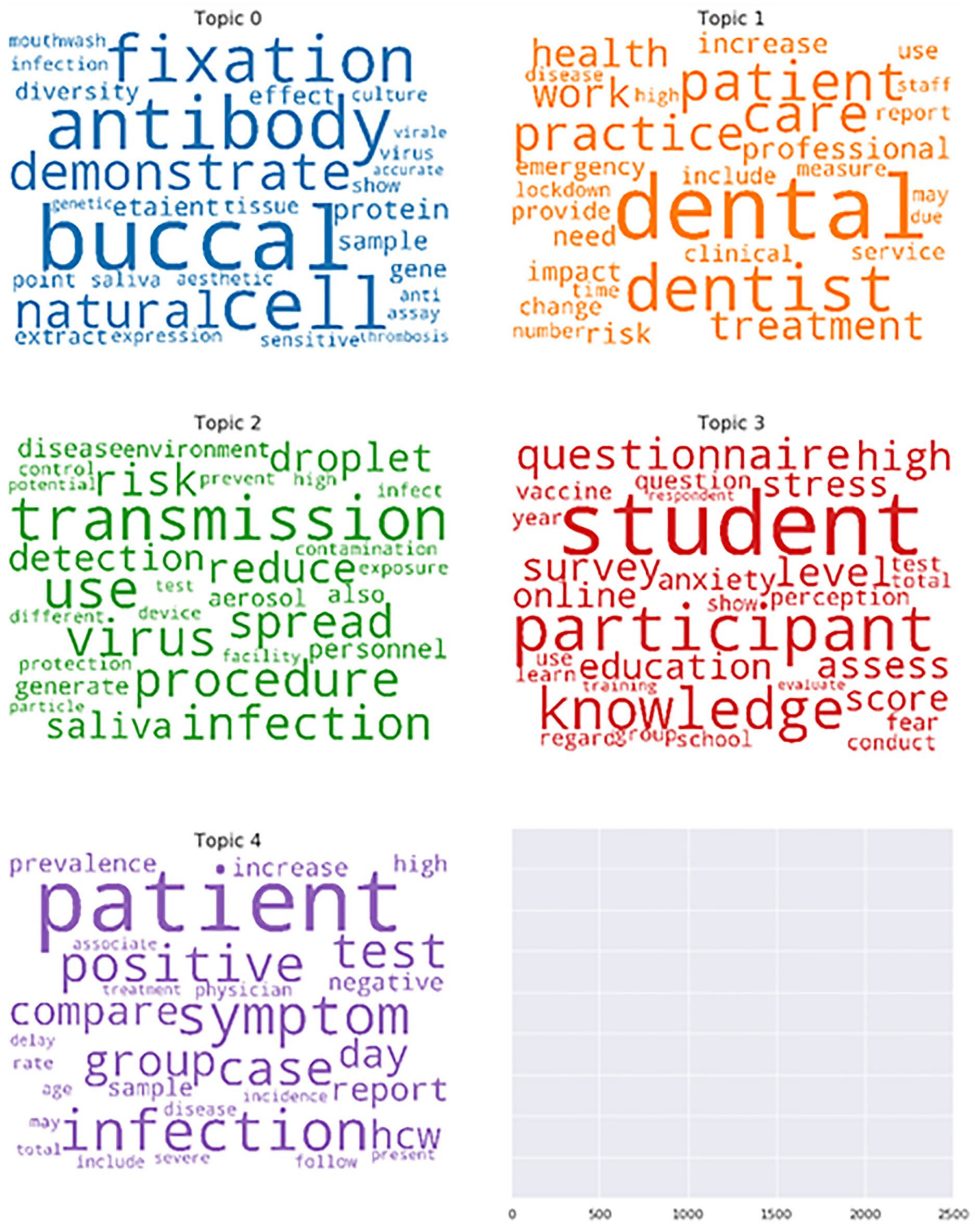
Word clouds of the five topics obtained after applying the Latent Dirichlet Allocation (LDA model). The font of the 30 more often words per topic reflects the decreasing frequency of appearance of each word.

After the first run of text mining applying the LDA model, 1,554 papers related to oral health/dentistry were classified into five topics (Fig. 2). The topics 0, 1, 2, 3 and 4 contained 68, 826, 185, 242 and 233 papers, respectively. The size of topic 1 remains between 700 and 800 documents in successive runs of the LDA model adjusted to generate 8 or 10 topics. Therefore, the initial classification of five topics was kept.

Table 1 shows the number of papers classified by topic and each topic's top 10 words or descriptive terms. According to the top 10 words, the five topic subjects were (0) the oral cavity as an entrance for the coronavirus into the body, (1) dental clinical practice and services offered during the pandemic, (2) the risk of COVID-19 transmission during clinical practice, (3) the psychological implications of COVID-19 for a dentist and the impact of COVID-19 in dental schools and education, (4) oral manifestations of COVID-19, oral hygiene and oral cancer. A multidimensional scaling analysis shows that topics 1 and 3 were the most similar.

**Table 1:** Top 10 keywords or descriptive terms per topic and number of articles per topic.

Topic	Keywords (descriptive terms)	n articles (%)
0	Buccal, antibody, cell, fixation, demonstrate, natural, protein, *etaient*, sample, effect	68 (4.38)
1	Dental, dentist, patient, care, practice, work, treatment, health, professional, risk	826 (53.15)
2	Transmission, use, virus, procedure, infection, spread, risk, reduce, droplet, detection	185 (11.9)
3	Student, participant, knowledge, questionnaire, high, level, survey, assess, education, stress	242 (15.57))
4	Patient, infection, symptom, positive, test, case, group, compare, day, hcw (healthcare workers)	233 (14.99)

### Agreement between text mining and author's review

The first round of text mining resulted in 1,554 papers that the authors reviewed by title, abstract and excerpt. The authors classified the papers into 17 categories based on the content (Table 2). Therefore, the five topics generated by the LDA model during the text mining analysis were divided into more specific subjects. The main categories obtained by the author's review were “Biosafety in dental practice” (n = 186, 17.4%) and “Dental practice during the pandemic” (n = 182, 17.0%).

**Table 2:** Description of the 17 categories obtained after the authors reviewed the title, abstract and excerpt. The count and frequency of articles per category are shown.

Category	n articles	Frequency %
Biosafety in dental practice	186	17.4
Dental practice during the pandemic	182	17.0
Psychological implications for dentists/patients	137	12.8
Dental education during the pandemic	134	12.5
Knowledge, Attitudes, and Practices against COVID-19	95	8.9
Dental treatments during the pandemic	88	8.2
Risk of COVID-19 transmission in dental practice	53	5.0
Economic implications of the pandemic on dental practice	36	3.4
Modifications in oral hygiene during the pandemic	32	3.0
Vaccination	23	2.2
COVID-19 detection in saliva	22	2.1
Mouth as a source of aerosols	20	1.9
Oral manifestations in patients with COVID-19	21	2.0
Oral diseases and risk of COVID-19	13	1.2
Oral microbiome in COVID-19 infection	8	0.7
Legal matters	4	0.4
Other	6	0.6
Total	1068	

Finally, the full text of 21 papers in the category “Oral manifestations in patients with COVID-19” was reviewed due to its clinical relevancy. This category is part of topic 4. A database was created to summarise the results of eight studies that passed the full-text review and contain the frequency of the oral manifestations identified in patients with COVID-19 (Table 3). In these eight studies, the most common oral manifestations were xerostomia (patient’s sensation of dry mouth) reported in six studies, burning mouth and mouth pain or swelling reported in five studies, impaired taste and ulcers in four studies, and dysphagia in three studies (Supplementary Table 2). The detection methods of the oral manifestations were clinical staff diagnosis and self-diagnosis. The two most frequent oral manifestations diagnosed by clinical staff were salivary gland ectasia (*n* = 46, 2.0%) and U-shaped lingual papillitis (*n* = 35, 1.5%). The most frequent oral manifestations identified by auto-diagnosis were xerostomia (*n* = 389, 17.1%) and mouth pain or swelling (*n* = 265, 11.7%) (Table 4).

**Table 3:** Characteristics of the included studies and the population evaluated.

Author (year)	Country	Study design	Study population (n)	Patients with oral alterations (n)	Male/female (%)	Age (mean or range)
Subramaniam et al*.*, 2021	India	Short-term observational study	713	9	58.3/41.7	12–80
Kady et al*.*, 2021	Egypt	Retrospective cohort study	58	39	53.4/46.6	18–46
Soares et al*.*, 2022a	Brazil	Retrospective cohort study	14	No report	71.4/28.6	22–88
González et al*.*, 2021)	Spain	Retrospective cohort study	666	78	No report	No report
Omezli and Torul, 2021	Turkey	Retrospective cohort study	107	No report	52.3/47.7	13–70
Gherlone et al*.*, 2021	Italy	Retrospective and prospective cohort study	122	31	75.4/24.6	53.9–74.1
Abubakr et al*.*, 2021	Egypt	Retrospective cohort study	573	410	28.8/71.2	19–50
Sinjari et al*.*, 2020	Italy	Retrospective cohort study	20	No report	55/45	35–91

**Table 4:** Oral manifestations identified in COVID-19 patients regarding the detection method.

Oral manifestation	Number of patients	Detection method	Study
Aphthous stomatitis	21	Detected by dentist/physician	^24^
Burning mouth	18	Detected by dentist/physician	^24,35^
	32	Self-diagnosis	^36–38^
Candidiasis	3	Detected by dentist/physician	^24^
Cheilitis	3	Detected by dentist/physician	^35^
Dysphagia	1	Detected by dentist/physician	^35^
	16	Self-diagnosis	^36,37^
Enanthema	2	Detected by dentist/physician	^24^
Erythema of tongue margins	1	Detected by dentist/physician	^30^
Extensive ecchymosis	2	Detected by dentist/physician	^35^
facial asymmetry	1	Detected by dentist/physician	^23^
Facial tingling	4	Detected by dentist/physician	^23^
Geographic tongue	1	Detected by dentist/physician	^35^
Gingival bleeding	4	Self-diagnosis	^36^
Glossitis with patchy Depapillation	12	Detected by dentist/physician	^24^
Halitosis	70	Self-diagnosis	^31^
Impaired taste	14	Detected by dentist/physician	^23^
	72	Self-diagnosis	^36–38^
Ischemic mucosa	3	Detected by dentist/physician	^39^
Lesions on the right side of the lower lip	1	Detected by dentist/physician	^35^
Lip necrosis	1	Detected by dentist/physician	^35^
Masticatory muscle weakness	23	Detected by dentist/physician	^23^
Mouth and/or lip spots	1	Detected by dentist/physician	^35^
	8	Self-diagnosis	^36^
Mucositis	15	Detected by dentist/physician	^24,35^
Pain or swellings	24	Detected by dentist/physician	^23,24^
	265	Self-diagnosis	^31,36,38^
Papillary atrophy	4	Detected by dentist/physician	^35^
Petechiae	5	Detected by dentist/physician	^39^
Pin pricking sensation on the back of her throat	1	Detected by dentist/physician	^35^
Reddish macule	3	Detected by dentist/physician	^39^
Salivary gland ectasia	46	Detected by dentist/physician	^23^
Smell alteration	12	Detected by dentist/physician	^23^
	23	Self-diagnosis	^38^
TMJ abnormalities	9	Detected by dentist/physician	^23^
Tongue redness	5	Self-diagnosis	^36^
Ulcers	12	Detected by dentist/physician	^35,39^
	146	Self-diagnosis	^36,39^
U-shaped lingual papillitis	35	Detected by dentist/physician	^24^
Vesicles	3	Detected by dentist/physician	^35,39^
White tongue	5	Detected by dentist/physician	^24^
Xerostomia	16	Detected by dentist/physician	^35,36^
Xerostomia	389	Self-diagnosis	^31,36–38^

## Discussion

The COVID-19 pandemic has profoundly impacted society, not only on a health level but also on social aspects such as economics and education. Lockdown measures to contain the COVID-19 spread led to the closure of businesses, schools, commercial stores, and government offices. The practice and teaching of Odontology have been affected during the pandemic due to the high risk of exposure to SARS-CoV-2^18–20^. Cancellation of most dental appointments during the pandemic, except for emergencies, and the astringent safety measures that odontologists must have taken to resume patient care during economic reactivation affected odontology worldwide. Virtual classes, fewer patients in clinics, and the inability to conduct research with patients were challenges for dental schools^21^.

Due to the clinical relevancy, we selected the topic “Oral manifestations in patients with COVID-19”, among 17 categories, for a deeper analysis. The two most common oral alterations in patients with COVID-19 diagnosed by clinical staff were salivary gland ectasia and U-shaped lingual papillitis. The main oral manifestations identified by auto-diagnosis were xerostomia and mouth pain or swelling. Early infection of the salivary glands by SARS-CoV-2 may be associated with salivary gland ectasia. This hyperinflammation of the salivary gland is significantly related to protein C-reactive and LDH levels, both of which are COVID-19 severity biomarkers^22^. Patients’ evaluation has shown that salivary gland ectasia is associated with a more severe course of COVID-19^23^. U-shaped lingual papillitis is an inflammation of the tongue’s papillae. It could be caused by direct inflammation or drying of the oral mucosa or poor oral health^24^.

The presence of xerostomia in patients with COVID-19 has been reported previously^25–27^. Xerostomia is a sign of dehydration, which can occur secondary to infections^28^. Early infection of the salivary glands by the SARS-CoV-2, affecting their function and leading to changes in the flow and composition of saliva, is one possible cause of xerostomia in the early stages of COVID-19^26^. It has been hypothesised that SARS-CoV-2’s neuroinvasive and neurotropic potential causes xerostomia, as it can enter the nervous system via angiotensin-converting enzyme 2^29^. It is also postulated that dysgeusia, a common manifestation of COVID-19, may be caused by xerostomia because changes in the amount and composition of saliva can cause gustatory dysfunction^30^. Xerostomia in patients with COVID-19 appears 1 or 2 days before other symptoms and may be helpful for early diagnosis and treatment implementation, preventing virus transmission.

The second most frequent oral manifestation by auto-diagnosis was mouth pain and swelling. The studies reviewed showed that patients primarily experienced pain or swelling in the salivary glands, jaw, or chewing. The muscles were the primary source of the oral pain. This is consistent with the observation that 76.4% of patients with COVID-19 had myalgia. They may feel muscle pain in the upper and lower jaw. Another source of pain can be headaches (70% of patients). Headache stimulates the trigeminal nerve, causing the release of neuropeptides that cause blood vessel dilation, inflammation, and pain^13–17,31–34^.

The data mining strategies, such as the one applied here, help analyse a vast amount of information; however, they require a human understanding of the keyword combinations and information printed by the method to avoid missing relevant documents or including thousands of irrelevant articles to reach the aim of the study.

The identification of 17 categories or subjects reflects the variety of themes covered within a single paper; for example, an article can focus on biosafety, psychological repercussions, and epidemiology simultaneously. When an article contains more than one paper, the LDA model assigns the document to more than one topic, although one of these topics may dominate the others. Therefore, the topics can overlap as topics 1 and 3 do in the present study. Another consequence of the subject variety within a paper is that each topic obtained by the model included more than one subject. For example, topic 4 was related to oral manifestations of COVID-19, oral hygiene and oral cancer.

Zengul et al. (2021) use a text mining approach to classify the NIH COVID-19 Portfolio as of November 2021 based on abstracts and titles. They identified 11 major research areas (topics), including “Epidemiologic Modelling”, “Mechanism of Disease”, “Protection/Prevention”, “Mental/Behavioural Health” and “Detection/Testing”. Additionally, they found that only five of the 11 abstract-based topics had a significant correlation with title-based topics and recommended revising the use of titles as the first step in developing an evidence-based medicine analysis portfolio^10^. None of the topics covered by Zengul et al. was related to oral health or dentistry.

Our analysis also reveals a challenge in selecting and classifying papers based on titles, abstracts, or excerpts, as it is common in systematic reviews and evidence-based medicine/dentistry. To build the corpus of text mining analysis in dentistry, we suggest using keywords or MeSH terms as the first filter of articles. The limitations of this study included a unique source of information; the literature analysis was based on the COVID-19 Open Research Dataset (CORD-19) database.

A perspective of this study is to apply text mining tools to identify the keywords that distinguished the 17 categories generated by the “author's review” and use those keywords as the basis for applying machine learning algorithms to construct a predictive model that can improve the selection and classification of the papers, reducing the time and necessity of curation of the documents.

## Conclusions

Using text mining to identify oral health/dentistry-related articles in a large dataset of COVID-19 publications, we found that patients with COVID-19 experienced a wide range of oral changes. The most common oral manifestations were those affecting the salivary glands, such as xerostomia or salivary gland ectasia, followed by oral pain and inflammation.

Text mining is a helpful tool for analysing and sorting massive document datasets. However, it was necessary to combine text mining with the author´s review by title, abstract and excerpts to avoid the loss of data or the inclusion of unnecessary information.

### Supplementary Information

Supplementary Table 1.

Supplementary Table 2.

## Data Availability

This published article and its supplementary information files include all data generated or analysed during this study.

## References

[1] Zhang J, Dong X, Liu G, Gao Y (2023). Risk and protective factors for COVID-19 morbidity, severity, and mortality. Clin Rev Allergy Immunol.

[2] Dziewas R, Warnecke T, Zürcher P, Schefold JC (2020). Dysphagia in COVID-19 –multilevel damage to the swallowing network?. Eur J Neurol.

[3] Farshidfar N, Hamedani S (2021). Hyposalivation as a potential risk for SARS-CoV-2 infection: Inhibitory role of saliva. Oral Dis.

[4] Pang W (2020). Tongue features of patients with coronavirus disease 2019: a retrospective cross-sectional study. Integr Med Res.

[5] Pollard CA, Morran MP, Nestor-Kalinoski AL (2020). The COVID-19 pandemic: A global health crisis. Physiol Genom.

[6] Wang, L. L. *et al.* CORD-19: The COVID-19 Open Research Dataset. Preprint at https://arxiv.org/abs/2004.10706v4 (2020).

[7] Tandan M, Acharya Y, Pokharel S, Timilsina M (2021). Discovering symptom patterns of COVID-19 patients using association rule mining. Comput Biol Med.

[8] Ertek, G., Tapucu, D. & Arin, I. Text Mining with RapidMiner. in *RapidMiner Data Mining Use Cases and Business Analytics Applications.* (Eds. Hofmann, M. & Klinkenberg, R.) 518 (Chapman & Hall, 2013).

[9] Reddy S (2021). Use and validation of text mining and cluster algorithms to derive insights from Corona Virus Disease-2019 (COVID-19) medical literature. Comput Methods Programs Biomed Updat.

[10] Zengul FD (2021). A critical analysis of COVID-19 research literature: Text mining approach. Intell Based Med.

[11] Srinivasa-Desikan, B. *Natural Language Processing and Computational Linguistics: A practical guide to text analysis with Python, Gensim, spaCy, and Keras*. (Ed Coutinho S.) 286 (Packt, 2018).

[12] Blei DM, Ng AY, Jordan MI (2003). Latent dirichlet allocation. JMLR.

[13] Badran Z, Gaudin A, Struillou X, Amador G, Soueidan A (2020). Periodontal pockets: A potential reservoir for SARS-CoV-2?. Med Hypotheses.

[14] Berton F (2021). Dental calculus—A reservoir for detection of past SARS-CoV-2 infection. Clin Oral Invest.

[15] Brandão TB (2020). Oral lesions in SARS-COV-2 infected patients: Could the oral cavity be a target organ?. Oral Surg Oral Med Oral Pathol Oral Radiol.

[16] Carreras-Presas CM, Sánchez JA, López-Sánchez AF, Jané-Salas E, Pérez MLS (2021). Oral vesiculobullous lesions associated with SARS-CoV-2 infection. Oral Dis.

[17] Rosa GRML, Libra M, Pasquale RD, Ferlito S, Pedullà E (2021). Association of viral infections with oral cavity lesions: Role of SARS-CoV-2 infection. Front Med.

[18] Elzein R (2021). Legal liability facing COVID-19 in dentistry: Between malpractice and preventive recommendations. J Forensic Leg Med.

[19] Chowdhry A, Kapoor P, Kharbanda OP, Popli DB (2021). Saliva and COVID 19: Current dental perspective. J Oral Maxillofac Pathol.

[20] Kapoor P (2021). Exploring salivary diagnostics in COVID-19: A scoping review and research suggestions. BDJ Open.

[21] Rolland F (2022). Impact of the first wave of the COVID-19 pandemic on French Health students. Encephale.

[22] Zangrillo A (2020). Characteristics, treatment, outcomes and cause of death of invasively ventilated patients with COVID-19 ARDS in Milan. Italy. Critical Care Resusc.

[23] Gherlone EF (2021). Frequent and persistent salivary gland ectasia and oral disease after COVID-19. J Dent Res.

[24] González AN (2021). Are oral mucosal changes a sign of COVID-19? A cross-sectional study at a field hospital. Actas Dermosifiliogr.

[25] Fantozzi PJ (2020). Xerostomia, gustatory and olfactory dysfunctions in patients with COVID-19. Am J Otolaryng.

[26] Fathi Y, Hoseini EG, Atoof F, Mottaghi R (2021). Xerostomia (dry mouth) in patients with COVID-19: A case series. Future Virol.

[27] Silva LDCME (2022). Xerostomia prevalence in COVID-19 patients a rapid systematic review. Oral Surg Oral Med Oral Pathol Oral Radiol.

[28] Saniasiaya J (2020). Xerostomia and COVID-19: Unleashing Pandora’s Box. Ear Nose Throat J.

[29] Freni F (2020). Symptomatology in head and neck district in coronavirus disease (COVID-19): A possible neuroinvasive action of SARS-CoV-2. Am J Otolaryng.

[30] Liu L (2011). Epithelial cells lining salivary gland ducts are early target cells of severe acute respiratory syndrome coronavirus infection in the upper respiratory tracts of rhesus macaques. J Virol.

[31] Abubakr N, Salem ZA, Kamel AHM (2021). Oral manifestations in mild-to-moderate cases of COVID-19 viral infection in the adult population. Dent Medical Problems.

[32] Fukuda K (2016). Diagnosis and treatment of abnormal dental pain. J Dent Anesthesia Pain Medicine.

[33] Campus G (2021). The COVID-19 pandemic and its global effects on dental practice. An international survey. J Dent.

[34] Negucioiu M, Bucur A, Lucaciu O, Soanca A, Roman A (2020). Management of SARS-CoV-2 transmission in emergency dental settings: Current knowledge and personal experience. Disaster Med Public.

[35] Subramaniam T, Nikalje M, Jadhav S (2021). Oral manifestations among COVID-19: An observational study of 713 patients. Dent Res J.

[36] El Kady DM (2021). Oral manifestations of COVID-19 patients: An online survey of the Egyptian population. Clin Exp Dent Res.

[37] Sinjari B (2020). SARS-CoV-2 and oral manifestation: An observational, human study. J Clin Medi.

[38] Omezli MM, Torul D (2021). Evaluation of the xerostomia, taste and smell impairments after Covid-19. Med Oral Patol Oral Cir Bucal.

[39] Soares CD (2022). Oral manifestations of coronavirus disease 2019 (COVID-19). Am J Surg Pathol.

