# Supplementary material for: Oral manifestations in patients with coronavirus disease 2019 (COVID-19) identified using text mining: an observational study
Source: Sci Rep. 2023 Oct 18;13:17770. doi: 10.1038/s41598-023-44784-2 (PMC10584950; doi:10.1038/s41598-023-44784-2)
Supplement: Supplementary file 2 — Supplementary Table 2. [file 41598_2023_44784_MOESM2_ESM.docx]

**Supplementary Material**

**Oral manifestations in patients with coronavirus disease 2019 (COVID-19) identified using text mining: an observational study.**

Sandra Guauque-Olarte^1^, Laura Cifuentes-C^2^, Cristian Fong^3^

^1^Faculty of Dentistry, Universidad Cooperativa de Colombia campus Envigado, Colombia. sandra.guauque@campusucc.edu.co

^2^Faculty of Dentistry, Universidad Cooperativa de Colombia campus Pasto, Colombia. laura.cifuentesc@campusucc.edu.co

^3^Faculty of Medicine, Universidad Cooperativa de Colombia campus Santa Marta, Colombia. cristian.fongr@campusucc.edu.co

*Corresponding author:

Sandra Guauque-Olarte, MSc, PhD

Assistant Professor

Faculty of Dentistry

Universidad Cooperativa de Colombia

Address: Cra. 47 No. 37 sur 18

Envigado, Antioquia, Colombia

Tel: (57)3016491638

sandra.guauque@campusucc.edu.co

**Supplementary Table 2.** The oral manifestations reported in the articles with their number and frequency.

| **Reference** | **Oral manifestation** | **n** | **Frequency (%)** |
| --- | --- | --- | --- |
| Subramaniam et al, 2021 | Ulcers | 4 | 0.56 |
|  | Papillary atrophy | 4 | 0.56 |
|  | Mucositis | 3 | 0.42 |
|  | Cheilitis | 3 | 0.42 |
|  | Xerostomia | 3 | 0.42 |
|  | Burning mouth | 2 | 0.28 |
|  | Mouth and/or lip spots | 1 | 0.14 |
|  | Vesicles | 1 | 0.14 |
|  | Lip necrosis | 1 | 0.14 |
|  | Geographic tongue | 1 | 0.14 |
|  | erythema of tongue margins | 1 | 0.14 |
|  | lesions on the right side of the lower lip | 1 | 0.14 |
|  | pin pricking sensation on the back of her throat | 1 | 0.14 |
| Kady et al, 2021 | Dysphagia | 1 | 0.14 |
|  | Impaired taste | 52 | 89.66 |
|  | Xerostomia | 23 | 39.66 |
|  | Pain or swellings | 14 | 24.14 |
|  | Dysphagia | 13 | 22.41 |
|  | Burning mouth | 13 | 22.41 |
|  | Ulcers | 10 | 17.24 |
|  | Mouth and/or lip spots | 8 | 13.79 |
|  | tongue redness | 5 | 8.62 |
| Soares et al, 2022 | Gingival bleeding | 4 | 6.90 |
|  | Ulcers | 8 | 57.14 |
|  | Petechiae | 5 | 35.71 |
|  | Ischemic mucosa | 3 | 21.43 |
|  | Reddish macule | 3 | 21.43 |
|  | Vesicles | 2 | 14.29 |
| Sinjari et al, 2020 | Extensive ecchymosis | 2 | 14.29 |
|  | Xerostomia | 6 | 30 |
|  | Impaired taste | 5 | 25 |
|  | Burning mouth | 4 | 20 |
|  | Dysphagia | 3 | 15 |
| González et al, 2021 | U-shaped lingual papillitis | 35 | 5.26 |
|  | Pain or swellings | 20 | 3.00 |
|  | Glossitis with patchy depapillation | 12 | 1.80 |
|  | aphthous stomatitis | 21 | 3.15 |
|  | Mucositis | 12 | 1.80 |
|  | Burning mouth | 16 | 2.40 |
|  | Candidiasis | 3 | 0.45 |
|  | Enanthema | 2 | 0.30 |
|  | White tongue | 5 | 0.75 |
| Omezli and Torul, 2021 | Xerostomia | 43 | 40.2 |
|  | Smell alteration | 23 | 21.5 |
|  | Pain or swellings | 18 | 16.8 |
|  | Burning mouth | 15 | 14.0 |
|  | Impaired taste | 15 | 14.0 |
| Gherlone et al, 2021 | Salivary gland ectasia | 46 | 38.0 |
|  | Masticatory muscle weakness | 23 | 19 |
|  | Impaired taste | 14 | 17 |
|  | Smell alteration | 12 | 14 |
|  | Xerostomia | 13 | 10.7 |
|  | TMJ abnormalities | 9 | 7 |
|  | Facial tingling | 4 | 3 |
|  | Pain or swellings | 4 | 3 |
|  | facial asymmetry | 1 | 0.8 |
| Abubakr et al, 2021 | Xerostomia | 317 | 47.6 |
|  | Pain or swellings | 233 | 35 |
|  | Ulcers | 136 | 20.4 |
|  | Halitosis | 70 | 10.5 |
